# The Synergistic Effect of WS_2_ and SWNTs on Tribological Performance of Polyether MDI Polyurethane Elastomer under Dry and Wet Friction Conditions

**DOI:** 10.3390/nano12081267

**Published:** 2022-04-08

**Authors:** Gang Lu, Changgeng Shuai, Yinsong Liu, Xue Yang, Xiaoyang Hu

**Affiliations:** 1 Institute of Noise and Vibration, Naval University of Engineering, Wuhan 430033, China; chgshuai@163.com (C.S.); liuyinsong1991@163.com (Y.L.); huxy_nue@163.com (X.H.); 2 Key Laboratory of Ship Vibration and Noise, Wuhan 430033, China

**Keywords:** polyurethane elastomer, WS_2_, SWNTs, synergistic effect, tribological performance

## Abstract

To adapt to the complex application of polyurethane bearings, it is feasible to improve the tribological performance of single polyurethane-based friction materials through the synergistic effect produced by multi-component-lubricating fillers. In this context, rather than using tungsten disulfide (WS_2_), which has demonstrated excellent self-lubricating performance as a lubricating oil additive, this paper proposes that WS_2_ and single-walled carbon nanotubes (SWNTs) can be designed for addition into a polyether 4,4′-diphenylmethane diisocyanate (MDI) polyurethane matrix as self-lubricating fillers so as to explore the synergistic effect of micro- and nano-lubricating fillers on the tribological performance of polyurethane matrix materials. Through a series of characterizations and tests, it was found that the dispersion of two-component-lubricating additives in a polyurethane matrix is improved when the ratio of WS_2_ to SWNTs is roughly 2:1. In this case, the tribological performance of polyurethane matrix composites is more satisfactory than at other ratios. In addition, compared with the blank sample, the tribological performance of the synergistically modified polyurethane composites under dry friction is more significantly improved with the increase in contact load, while there is no significant improvement under water lubrication. Aside from contributing to the idea of exploring the synergistic effect of WS_2_ and other micro or nanofillers, this method also opens up the possibility of practically applying WS_2_ in the field of friction.

## 1. Preface

Since the middle of the 20th century, polyurethane elastomer has been widely applied in various industrial settings because of its effective molecular design, high mechanical strength and strong environmental robustness [1,2,3]. For example, in the field of bearing, polyurethane material is referred to as “wear-resistant rubber.” Among various elastomer materials, it performs best in wear resistance. Compared to natural rubber, its wear resistance can be two to ten times better. At the same time, it also possesses high mechanical strength. In addition, it demonstrates excellent performance in vibration damping and cushioning, which makes it suitable for use in marine bearings and vibration damping components [4,5]. In recent years, with increasing complexity in the application of polyurethane-based materials on bearings, the use of a single polyurethane material cannot meet the needs of practical applications, such as the heat dissipation of polyurethane-based bearings in the context of dry friction [6] and the high friction coefficient caused by an excessive amount of impurities in the lubricating medium under the context of wet lubrication [7]. Thus, it is necessary to meet the requirements of environmental and working conditions through modification.

In general, there are two research perspectives on how to modify polyurethane friction materials. One is to introduce specific functional groups or chain segments to improve their tribological performance through the excellent molecular design of polyurethane soft and hard segments [8]. The other is to make use of micro and nanofillers, relying on the small size effect of nanoparticles to separate from the friction on contact surfaces [9] and improve the tribological performance of polyurethane composites. Given the requirement of operability and practicability, the latter is considered better.

Among a variety of wear-resistant and drag-reducing fillers, WS_2_ is extensively used as a lubricant due to its excellent anti-wear and self-lubricating properties exhibited under various, extreme working conditions [10]. The material presents itself as a gray, fine crystal or powder with a metallic luster. Due to an easily separable, layered structure, it shows similar lubricating properties to graphite. In addition, the performance of WS_2_ is better than that of molybdenum disulfide, with the friction coefficient reaching as low as 0.03. As a kind of material with excellent electrical and mechanical properties and thermal conductivity, SWNTs can be regarded as the crimping of single-layer graphene. For specific varieties of polymer matrix materials, a small amount of single-walled carbon nanotubes can be used to significantly improve the comprehensive properties of composites compared to traditional micro and nanofillers [11,12,13]. According to the relevant literature, there are few references made to the addition of WS_2_ and SWNTs additives to prepare the polyurethane friction composites intended to work together in the context of polyurethane friction. Therefore, polyurethane composites are studied in this paper through the synergistic blending of WS_2_ and SWNTs, with polyether MDI polyurethane elastomer as matrix friction material [14], which is essential for research on the synergistic effect of lubricating additives for friction materials.

## 2. Materials’ Design and Preparation

### 2.1. The Main Raw Materials

The main chemical materials used in this study included polyether MDI polyurethane prepolymer (MDI-PUP) with isocyanate group (NCO) content 13.25–13.65 wt.%; polyether polyol (PA), chemical purity with relative molecular weight (i.e., 2000); butanediol (BDO), analytical purity; SWNTs with an outside diameter of 1~2 nm and length of 5~30 μm, purity > 90 wt%, specific surface area > 380 m^2^/g (Zhongke Shidai Naneng Technology Co., Ltd., Chengdu, China) and WS_2_, with a size of 2 μm, mesh number of about 6500 and purity of 99.9% (Aladdin Biochemical Technology Co., Ltd., Shanghai, China).

### 2.2. Formulation Design of Friction Samples

Table 1 shows the formula design of polyurethane-based friction composites.

### 2.3. High-Speed Shear Mixing Device

**Figure 1:**
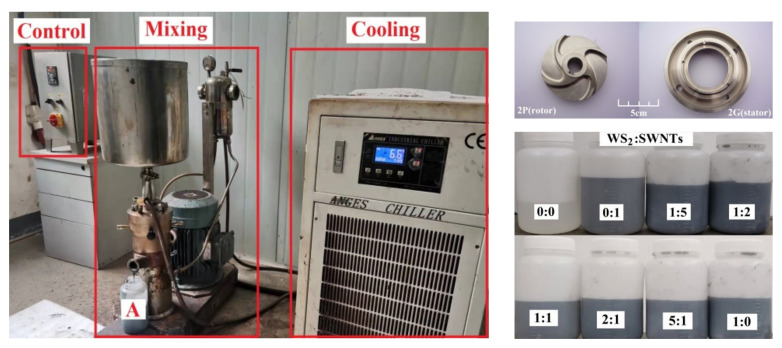
Self-made high-speed shear mixing device and component A after mixing.

As the key to the dispersion of two-component micro- or nano-lubricating fillers in polyurethane materials [15], dispersion technology plays a significant role in the preparation of composites. Therefore, it is proposed in this paper that micro- or nano-lubricating filler can be distributed in polyurethane prepolymer using self-made high-speed shear mixer to obtain material A for the subsequent process to be conducted. The high-speed shear mixer consists of three parts, as shown in Figure 1: control part, mixing part and cooling part. Among them, the stator and rotor dispersion head of the mixer is shown in the upper right figure, and the obtained component A is shown in the following figure on the right.

### 2.4. Experimental Procedure

(1) Prepare materials by complying with the formula in Table 1, and place the mold release agent in different molds in a 100 °C environment.
(2) Subsequently, pre-mix the measured MDI-PUP and fillers at the temperature of 70 °C, place them into a shear mixer and regulate at the rotational speed of 5000 r/min for deep mixing. Label the mixture as material A (Figure 1). Moreover, mix the measured PA and BDO evenly at 43 °C, and label the mixture as material B.
(3) Mix materials A and B. Then, place the mixture in a vacuum oven to remove the bubbles. Next, transfer the mixture to various molds and mature at 100 °C for 18 h. Finally, eject the samples (WSS_0-0_~WSS_1-0_) and place them in the ambient temperature for one week for further tests.

**Figure 2:**
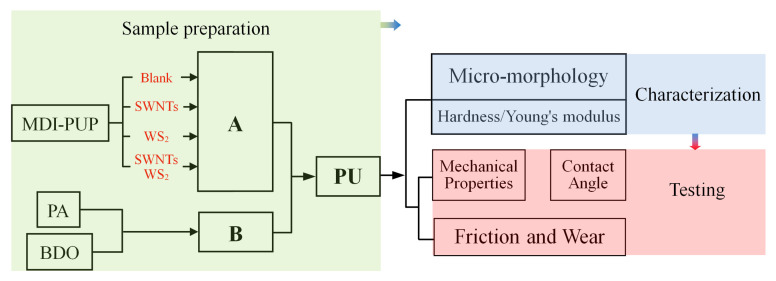
The design idea and research content of this paper.

The experimental process is illustrated in Figure 2.

## 3. Characterization and Test Analysis of Materials

### 3.1. Test Methods and Instruments

Table 2 details the characterization and test equipment information involved in this paper.

**Figure 3:**
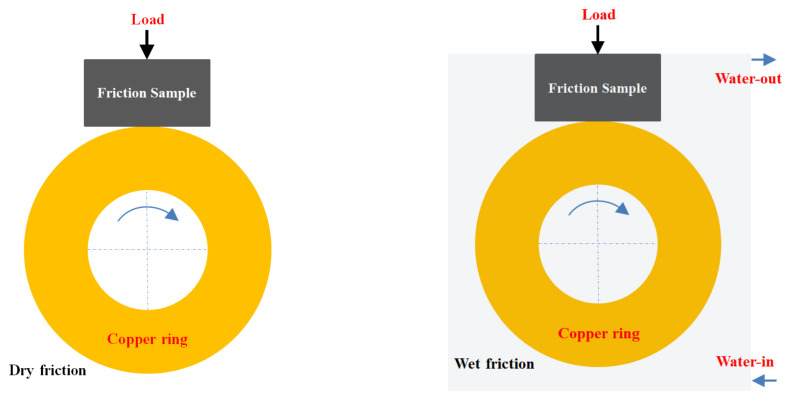
Schematic diagram of dry and wet friction test.

The design of the friction and wear test conducted using the above instruments is shown in Figure 3.

### 3.2. Morphology and Hardness Distribution

**Figure 4:**
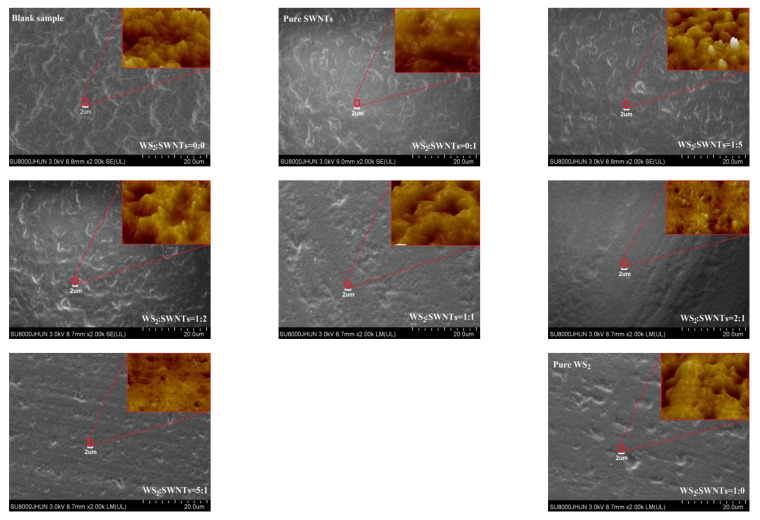
Surface morphology of WSS_0-0_ to WSS_1-0_ under electron and atomic force microscopes.

The surface morphology of polyurethane matrix composites is shown in Figure 4. The overall image is an image magnified by 2000 times under an electron microscope, while the upper right corner is a locally magnified atomic force image from a microscope.

According to the figure, with no addition of WS_2_ or SWNTs into the polyurethane material, the surface morphology of the material shows regularity. However, when WS_2_ and SWNTs are added, the surface of the composite shows agglomeration to different extents, which is attributed to the overly large, specific surface area of micro and nanoparticles. When distributed in the polyurethane material, small particles tend to aggregate with particles that have a smaller, specific surface area; as a result, they can maintain stability. In addition, this form of agglomeration will be exacerbated when the van der Waals forces between micro and nanoparticles exceed their own gravity [16,17]. Among them, the surface of the composite shows an improved level of regularity when WS_2_:SWNTs are 2:1 and 5:1. This is because the same components tend to agglomerate with the addition of micro and nano WS_2_ and SWNTs into polyurethane in a free state. However, the dispersion effect between components is improved due to the high-speed shear force that not only increases the entropy of the blend system, but also makes the two lubricating additives show a staggered arrangement. In addition, it is possible for a continuous network structure to develop between WS_2_ and SWNTs, which may lead to the synergy between nanofillers [18].

**Figure 5:**
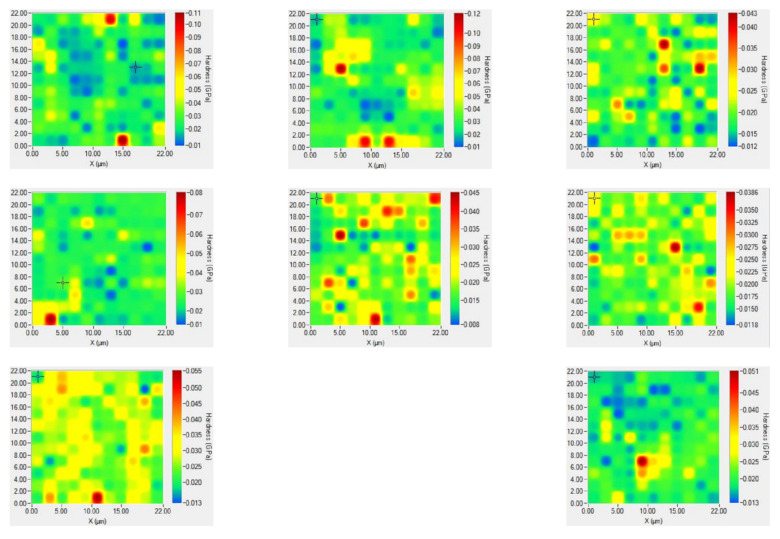
Reduced hardness distribution of WSS_0-0_ to WSS_1-0_ samples under nano indentation.

Figure 5 shows the Brinell hardness distribution of polyurethane composites combined with the hardness scale on the right of each figure. The difference in the hardness distribution of WSS_2-1_ is found to be least significant when WS_2_:SWNTs is 2:1, which is consistent with what is shown in Figure 4. In contrast, the hardness difference between other polyurethane composites will reach an excessively high level due to the agglomeration of micro- and nano-lubricating fillers.

### 3.3. Result Analysis

#### 3.3.1. Tensile Property

**Figure 6:**
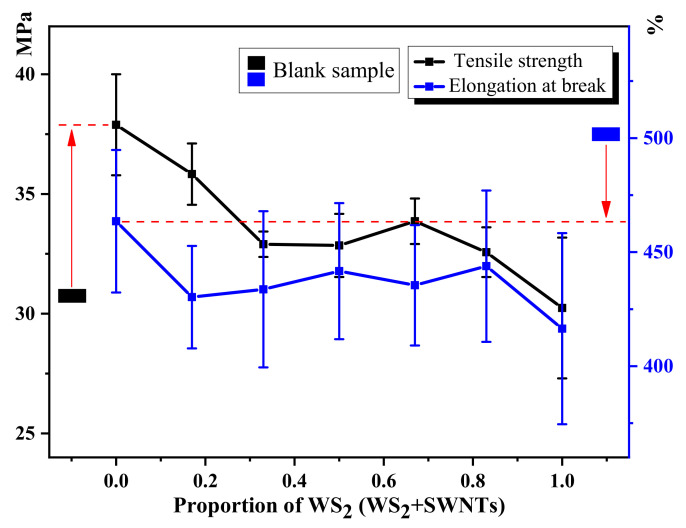
Tensile properties of polyurethane matrix friction composites.

Table 3 shows the tensile property data of polyurethane friction composites, which is used to draw Figure 6.

As shown in Figure 6, the addition of composite additives improves the tensile strength of polyurethane matrix composites to a significant extent. Comparatively, the addition ratio of WS_2_ and SWNTs affects the tensile strength of composites differently. In general, the degree of micro and nanofiller distribution in the matrix material has a considerable impact on the mechanical strength of the composite. The poor dispersion of fillers makes it easy for stress concentration to occur at the location where the fillers agglomerate [16], thus causing a significant reduction in the tensile strength and elongation of materials. According to the tensile strength curve shown in the figure, there is only a marginal improvement to the tensile properties of the composite when WS_2_:SWNTs is 2:1, which suggests that the distribution of the two fillers in the matrix is satisfactory at this ratio, as confirmed by Figure 4 and Figure 5. In addition, it can be seen from the results of Figure 4 and Figure 5 that the agglomeration in the composite is obvious when WS_2_ is added into the polyurethane matrix as a single lubricating additive.

#### 3.3.2. Contact Angle

**Figure 7:**
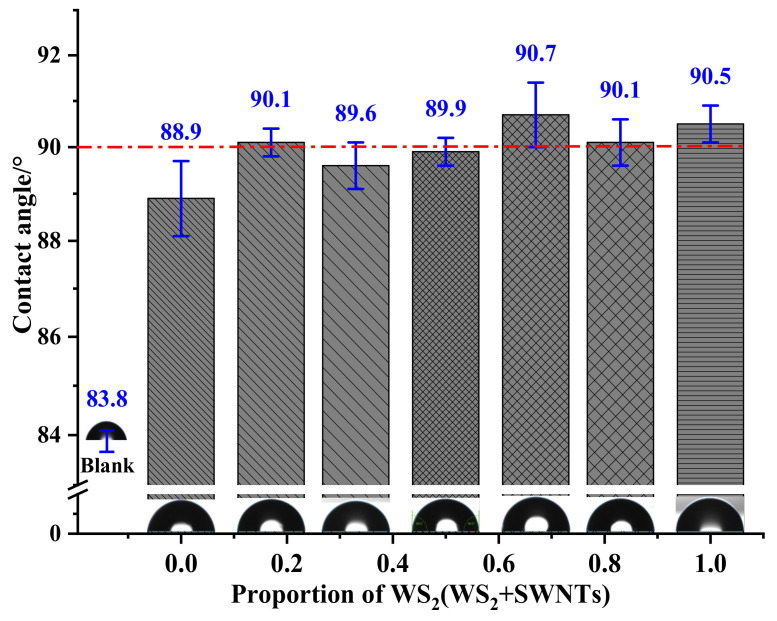
Water contact angle of polyurethane matrix friction composites.

Figure 7 shows the trend of changes in the water contact angle of WSS_0-0_~WSS_1-0_. According to this figure, the polyurethane without any micro- and nano-lubricating materials shows hydrophilicity. With the addition of WS_2_ and SWNTs into the polyurethane matrix, the water contact angle of the composite increases slightly and concentrates at about 90°. This is because the addition of WS_2_ and SWNTs with poor hydrophilicity leads to a slight increase in the water contact angle of the composites despite the hydrophilicity of polyether MDI polyurethane elastomer.

#### 3.3.3. Friction and Wear Properties

**Figure 8:**
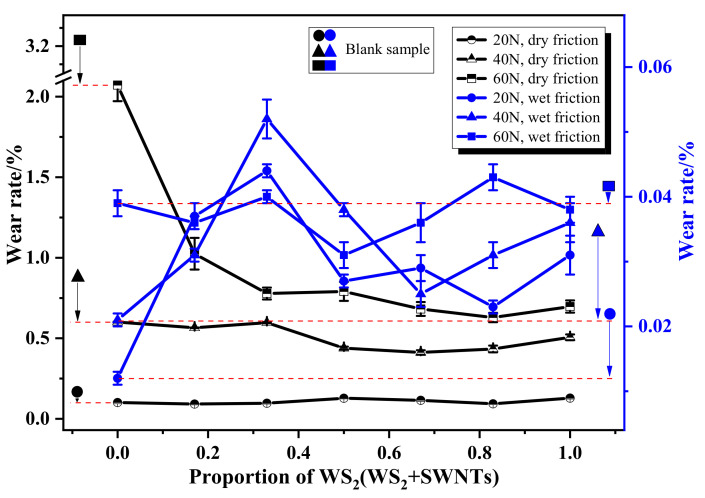
Wear rate of polyurethane matrix friction composites.

**Figure 9:**
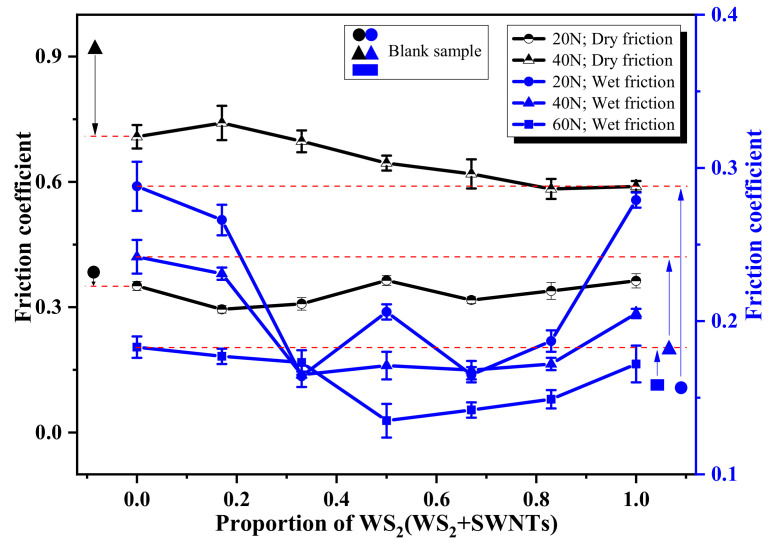
Friction coefficient of polyurethane matrix friction composites.

Table 4 lists the data on the tribological performance of polyurethane matrix composites, which were used to draw Figure 8 and Figure 9.

As shown in Figure 8, there is a significant variation in the wear rate of composites between dry friction and wet friction. To be specific, under the context of dry friction, the wear rate of the composites decreases slowly with an increase in the proportion of WS_2_, which is even more evident with the increase in contact load. When WS_2_:SWNTs reaches about 2:1, the wear rate is basically at its lowest. According to the analysis shown in Figure 4 and Figure 5, it is possible for WS_2_ and SWNTs to produce a satisfactory synergistic effect when WS_2_:SWNTs is about 2:1. As a result, the composite performs well in wear resistance. In addition, the improvement in the wear resistance of the composite under a heavy load is also related to the addition of a high proportion of WS_2_, which is because the accumulation of heat caused by high friction leads to the formation of a dense tungsten oxide (WO_3_) protective film [19], thus reducing the wear of the composite. Unlike the trend shown under the context of dry friction, the overall trend of changes in the wear rate of the composites under water lubrication is less significant, with excellent wear resistance shown by all of them. This is because the matrix material of the composites is hydrophilic polyurethane; despite the poor hydrophilicity of the added synergistic lubrication filler, the continuous phase structure formed by the polyurethane matrix can still cause the formation of continuous water film under water lubrication, which ensures the wear resistance of the composites.

As shown in Figure 9, in the context of dry friction, the friction coefficient of the polyurethane composite is significantly lower compared to the blank sample when the contact load increases. In contrast, in the context of wet friction, the friction coefficient of polyurethane composites shows no significant improvement compared to the blank sample. The friction coefficient ceases to increase only when WS_2_:SWNTs ranges from 1:2 to 5:1. This is because when the composite is in the dry friction state, the WS_2_ with a layered structure in the polyurethane composite shows excellent self-lubricating performance with the increase in contact load, while the lubricating effect is made increasingly evident with the increase in WS_2_ content. With the composite in the wet lubrication state, the addition of filler mitigates the water film lubrication effect, which can be explained by the increase in the contact angle of the polyurethane composite with the addition of WS_2_ and SWNTs, as shown in Figure 7. Therefore, the lubrication effect of the lubricating filler is manifested only when WS_2_ and SWNTs are evenly distributed. Consequently, the friction coefficient shows a continuous, declining trend when WS_2_:SWNTs is about 2:1. As suggested by the information shown in the figure, the above self-lubricating effect is more significant when the contact load increases, which is consistent with the pattern exhibited by the friction coefficient of the composite in the context of dry friction.

## 4. Conclusions

(1) Compared to a low contact load, the addition of WS_2_ improves the tribological performance of polyurethane composites under high load, whether in dry or wet conditions. Additionally, this effect is more significant with an increase in the proportion of WS_2_ in WS_2_ and SWNTs, which is attributed to the excellent anti-wear and self-lubricating properties of WS_2_ under high load.
(2) With the addition of WS_2_ and SWNTs, the higher the proportion of SWNTs, the more significant the improvement of the tensile strength for the composites. When the ratio of WS_2_ and SWNTs approaches 2:1, the distribution of composite additives in the polyurethane matrix is relatively satisfactory. Therefore, the tribological and tensile performance of polyurethane composites is generally acceptable.
(3) Due to the formation of a lubricating water film at the friction interface, the friction coefficient of the composites is reduced, which improves the hydrophilicity of the friction materials. This may also help improve the tribological performance.

## Figures and Tables

**Table 1:** Formulation design of polyurethane matrix friction composites.

Samples	A(phr)				B(phr)	
	MDI-PUP	WS_2_:SWNTs	WS_2_	SWNTs	PA	BDO
WSS_0-0_	100	0:0	0	0	30	12.8
WSS_0-1_		0:1	0	1.8		
WSS_1-5_		1:5	0.3	1.5		
WSS_1-2_		1:2	0.6	1.2		
WSS_1-1_		1:1	0.9	0.9		
WSS_2-1_		2:1	1.2	0.6		
WSS_5-1_		5:1	1.5	0.3		
WSS_1-0_		1:0	1.8	0		

thr: the number of parts by mass added to 100 parts by mass of rubber (or resin).

**Table 2:** Characterization and test equipment.

Instrument Name	Model	Remarks
Scanning electron microscope (SEM)	HITACHI Su8010	Resolution was 1.0 nm at 15 kV and 1.3 nm at 1 kV; electron gun was cold gun emission; accelerating voltage was 0.5~30 kV (0.1 kV/step).
Atomic force microscope (AFM)	NX-10	Resolution in XY direction was 0.001 nm and Z direction was 0.05 nm; scanning range in XY direction was 50 × 50 μm and Z direction was 15 μm.
Nanoindenter	Tl 980	Scanning area: 22 μm × 22 μm; The number of scanning points: 121.
Mechanical tester	MTS C42	Sample size: dumbbell shape
Contact angle tester	Fed-A3	Clean surface; Saterial size: 20 mm × 20 mm × 20 mm
Friction and wear tester	MRH-3A	Sample block size: 19 × 13 × 13 mm; copper ring size: Φ49.2 × 13.6 mm

**Table 3:** Tensile property data of polyurethane matrix friction composites.

Sample	Tensile /MPa	Maximum Error Limit /MPa	Elongation at Break /%	Maximum Error Limit /%
WSS_0-0_	30.73	1.59	501.96	16.82
WSS_0-1_	37.89	2.11	463.54	31.25
WSS_1-5_	35.83	1.28	430.26	22.47
WSS_1-2_	32.90	0.53	433.70	34.23
WSS_1-1_	32.85	1.32	441.67	29.84
WSS_2-1_	33.86	0.95	435.48	26.39
WSS_5-1_	32.57	1.04	443.84	33.17
WSS_1-0_	30.24	2.94	416.44	41.93

**Table 4:** Tribological data of polyurethane matrix composites under different working conditions.

Sample	20 N				40 N				60 N			
	Dry		Wet		Dry		Wet		Dry		Wet	
	w/%	f	w/%	f	w/%	f	w/%	f	w/%	f	w/%	f
WSS_0-0_	0.182	0.382	0.022	0.156	0.894	0.919	0.035	0.176	3.217	/	0.042	0.157
WSS_0-1_	0.101	0.351	0.012	0.288	0.601	0.708	0.021	0.242	2.072	/	0.039	0.183
WSS_1-5_	0.092	0.295	0.037	0.266	0.565	0.741	0.031	0.231	1.025	/	0.036	0.177
WSS_1-2_	0.097	0.308	0.044	0.164	0.597	0.697	0.052	0.165	0.778	/	0.040	0.173
WSS_1-1_	0.128	0.364	0.027	0.206	0.439	0.645	0.038	0.171	0.791	/	0.031	0.135
WSS_2-1_	0.115	0.317	0.029	0.165	0.412	0.619	0.025	0.168	0.682	/	0.036	0.142
WSS_5-1_	0.093	0.339	0.023	0.187	0.434	0.583	0.031	0.172	0.629	/	0.043	0.149
WSS_1-0_	0.129	0.363	0.031	0.279	0.506	0.589	0.036	0.205	0.697	/	0.038	0.172

w/%: Wear/%; f: Friction coefficient.

## Data Availability

The data presented in this study are available on request from the corresponding author.
